# Predicting wildfire ignition induced by dynamic conductor swaying under strong winds

**DOI:** 10.1038/s41598-023-30802-w

**Published:** 2023-03-10

**Authors:** Xinyue Wang, Paolo Bocchini

**Affiliations:** Department of Civil and Environmental Engineering, Catastrophe Modeling Center, ATLSS Engineering Research Center, Lehigh University, Bethlehem, 18015 USA

**Keywords:** Natural hazards, Governance, Civil engineering, Energy grids and networks

## Abstract

During high wind events with dry weather conditions, electric power systems can be the cause of catastrophic wildfires. In particular, conductor-vegetation contact has been recognized as the major ignition cause of utility-related wildfires. There is a urgent need for accurate wildfire risk analysis in support of operational decision making, such as vegetation management or preventive power shutoffs. This work studies the ignition mechanism caused by transmission conductor swaying out to nearby vegetation and resulting in flashover. Specifically, the studied limit state is defined as the conductor encroaching into prescribed minimum vegetation clearance. The stochastic characteristics of the dynamic displacement response of a multi-span transmission line are derived through efficient spectral analysis in the frequency domain. The encroachment probability at a specified location is estimated by solving a classical first-excursion problem. These problems are often addressed using static-equivalent models. However, the results show that the contribution of random wind buffeting to the conductor dynamic displacement is appreciable under turbulent strong winds. Neglecting this random and dynamic component can lead to an erroneous estimation of the risk of ignition. The forecast duration of the strong wind event is an important parameter to determine the risk of ignition. In addition, the encroachment probability is found highly sensitive to vegetation clearance and wind intensity, which highlights the need of high resolution data for these quantities. The proposed methodology offers a potential avenue for accurate and efficient ignition probability prediction, which is an important step in wildfire risk analysis.

## Introduction

Wildfire, also known as bushfire, can occur when there is unfavorable weather (low humidity, high temperature, high winds, etc.) combined with dry vegetation fuel. The past decades have witnessed wildfires becoming a severe threat around the world, such as in southern Europe, North America, and southeastern Australia^1–3^. Wildfires in different regions exhibit regional characteristics, as local conditions affect typical ignition causes, fire behavior, etc. In the United States, wildfire is a particularly important hazard in California, which has been suffering from frequent devastating wildfires. The California Department of Forestry and Fire Protection (CAL FIRE) reported a yearly average of 3217 wildfire incidents and 624,728 burnt acres in California during 2016~2020^4^. While wildfires can be started by a broad variety of causes (e.g., lightning, arson, smoking, etc.), electrical powerlines were shown to be the only non-declining ignition source^5^. Statistics showed that out of the top 20 most destructive California wildfires, at least five were started from power systems, including the 2018 Camp Fire, which destroyed 18,804 structures and claimed 85 lives^6^. In fact, power system-ignited incidents are more likely to develop into large wildfires, due to their special relationship with extreme weather conditions. A power network, comprising of numerous components and equipment, can experience a sharp increase in failure/faults under strong winds^7,8^. With the contribution of hot and dry air, various ignition mechanisms can be triggered, and fires may be started where combustible fuels are present. Moreover, strong winds can greatly facilitate the fire spread, while also hindering the firefighting efforts. Wind is the driving weather factor for powerline ignition. This has been indicated by the joint occurrence of powerline-related wildfires and seasonal extreme winds in California^9,10^. These foehn winds (known as Diablo winds or Santa Ana winds) are characterized by remarkable intensity and gustiness.

**Figure 1:**
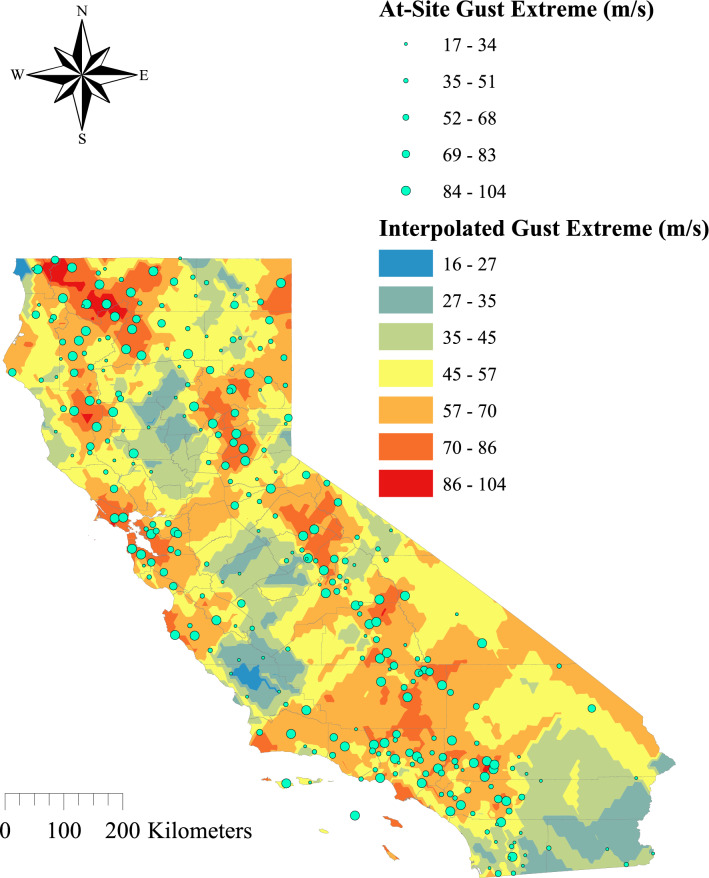
California wind hazard map (Return period = 20 years).

The electric power grid is generally considered to be composed of two systems: transmission system and distribution system. Compared to the distribution system, the transmission system plays a more critical role in power reliability because it transports bulk, high-voltage electricity over long distances. There is growing research interest in studying the reliability and resilience of power infrastructure subject to wind hazard^11–17^. In most cases, the structural failure of a certain component (e.g., transmission conductors, utility poles/towers) was studied. However, where wildfire is concerned, the relevant limit state is different from traditional structural failure, as emphasis is put on the probability of causing effective ignition mechanisms^18^. For instance, hot metal particles from conductor arcing and burning embers from conductor-vegetation contact are both eligible wind-induced failure modes, whereas structural failure (e.g., conductor rupture) is not necessarily dangerous^19^. It was shown that vegetation contact was the primary cause of power utility ignition in California, with a contribution of 53.5%^20^. Under high wind conditions, conductor-vegetation contact generally occurs in two forms: broken trees/limbs falling on the conductor (known as the “fall-in” issue), and conductor swinging out to nearby vegetation (known as the “grow-in” issue). Overhead transmission lines are usually supported by tall transmission towers, which makes the fall-in issue less likely. Instead, the vegetation grow-in issue is identified as a major threat to electric transmission systems^21^. Transmission conductors bear the greatest exposure to wind hazard events, as they span long distances across variable terrains. They are highly flexible, and large swinging displacements (10~20 m) can be observed around the mid-span^22^. It is anticipated that climate change will influence the magnitude and frequency of future extreme weather events. The American Society of Civil Engineers (ASCE) has been advocating adaptive infrastructure for a changing climate. As noted therein, potential adverse changes, such as prolonged dry seasons, warmer temperatures, and increased extreme wind intensities may worsen the situation of powerline-induced ignition^23,24^. One major challenge is to assess the impacts of climate change on built and natural systems based on climate projections. With varying complexities and goals, climate analysis may be carried out at different levels^24^. Even ignoring the future effects of climate change, a static wind hazard map with a 20-year return period was generated and it shows the severity of the problem (see Fig. 1). The methodology used for creating the wind hazard map is detailed in the Supplementary Information. Fig. 1 suggests that very strong winds, with a broad intensity range (17~104 m/s), are expected to occur in California. Distinct spatial variation can also be observed despite the sparsity of stations in some areas, which could pose a serious challenge on the operation of large-scale power grids.

In recognition of the potential of devastating wildfires started from power systems, California electric utilities are authorized to conduct preemptive Public Safety Power Shutoff (PSPS) in response to severe weather conditions^25^. In the fire season of 2019 alone, millions of people were affected by the rounds of power shutoffs, which lasted for more than one month^26,27^. Despite the immediate effectiveness in stopping power assets from causing fires, a PSPS event can lead to other significant disruptions, as communities and critical infrastructures are de-energized. Risk analysis is a powerful tool for decision making under uncertainties. In the PSPS context, two risks have to be balanced, namely the risk from utility-induced wildfires and the risk from events connected with the blackout, which can range from an increase of car accidents due to the lack of traffic lights, to health problems caused by shutting down domestic life-preserving equipment^28^. Wildfire risk analysis is generally concerned with three components: ignition probability, burn probability (or spread probability), and vulnerability^29^. In terms of ignition probability, some previous studies focused on developing statistical models by studying historical ignition records^30^. This purely data-driven type of approaches is versatile and applicable to various ignition sources. However, they are uninformative as to understanding the underlying failure and ignition mechanisms that could drive improvement measures and real-time decisions on PSPS. In contrast, there is a paucity of research work on wildfire ignition focusing on the physical interaction between high winds and electric power infrastructure, which is the focus of this study.

**Figure 2:**
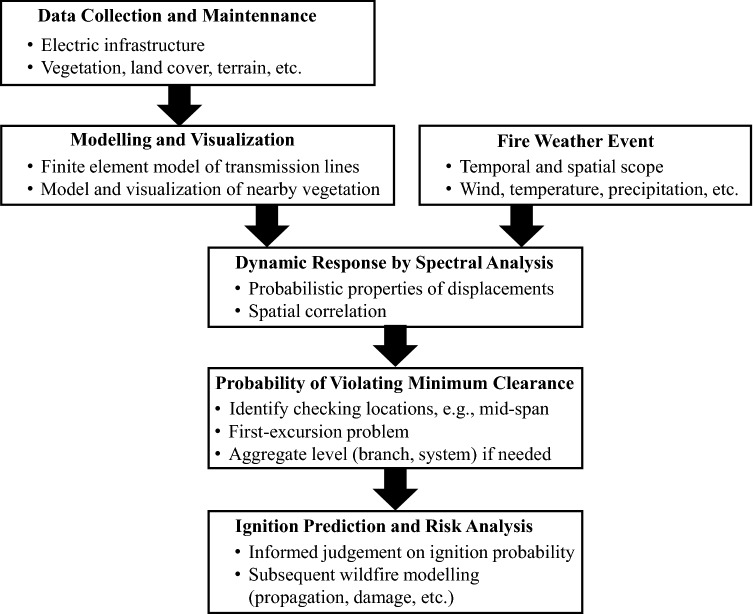
Framework of the proposed methodology (Note that the last box is provided for context but not included in the analysis).

The prediction of ignition has great influence on wildfire risk analysis because subsequent fire propagation simulation and fire damage analysis rely on ignition location and timing as input. Hence, this study focuses on the ignition due to the transmission conductor being blown close enough to the surrounding vegetation and causing flashover or sparks. Specifically, a methodology for estimating the probability of encroachment into baseline clearance (i.e., the initiating failure) is proposed, as summarized in Fig. 2.

The novelty of this paper is that it introduces the idea of studying the ignition-incurring clearance violation problem through a formal analysis of the structural responses, considering wind loading uncertainties. The encroachment probability computed using this methodology accounts for all the relevant factors, such as the duration of the wind event, the wind intensity, the transmission line (TL) properties, the vegetation clearance. It is worth noting that previous studies on the dynamics of conductor cables in the spectral domain (using a similar characterization for the wind stochastic process) focused on the stresses concerning the conductor failure. However, the application to vegetation encroachment entails a distinct focus on the conductor displacements. It thus requires that a new limit state equation and associated first-passage problem should be formulated, which is an original contribution of this work. The remainder of this paper is structured as follows. First, the background and practices of vegetation management are reviewed, following which the relevant limit state is defined. Second, the proposed methodology for computing the probability of encroachment is detailed. Finally, the application section gives two examples at different scales, where major findings are presented.

## Vegetation management for transmission systems

Vegetation growing near power infrastructure has long been recognized as a threat to the reliability of electric power networks, and it is particularly concerning in transmission systems. In fact, the shift of electrical current due to a failed TL may cause cascading failures elsewhere and cause massive power outages^31^. Meanwhile, urbanization has been driving power infrastructure into the wildland-urban interface (WUI), which is more forested and fire-prone, exacerbating the risks caused by the proximity to vegetation^9^. The vast majority of transmission lines use overhead conductors instead of underground cables, because the latter are much more expensive to install and maintain. As mentioned earlier, there are two types of vegetation-conductor interactions that can trigger failure: the fall-in type and the grow-in type. The fall-in failure mechanism involves substantial uncertainties on the vegetation side, including vegetation health condition, and fracture strength under wind loading, to name a few. Although modern technologies, such as Light Detection and Ranging (LiDAR)^32^ have facilitated vegetation data acquisition, the fall-in issue remains very difficult to predict, even in a probabilistic sense, given the complexity of vegetation and post-fracture windborne path. On the other hand, this paper focuses on the grow-in class of potential failures, which is closely related to the structural behavior. Specifically, the wind-induced dynamic displacement response of transmission lines is examined with the aim to better understand how it increases the probability of clearance encroachment, and in turn ignition. The corresponding ignition mechanism is the flashover (or sparkover) phenomenon, in which electrical current jumps through air from the conductor to a nearby object (typically trees). The energy released from the high-voltage current can result in ignition and even fires in the presence of low-moisture vegetation and dry atmosphere. It is important to note that flashover can occur even when there is no direct contact between conductor and trees.

**Figure 3:**
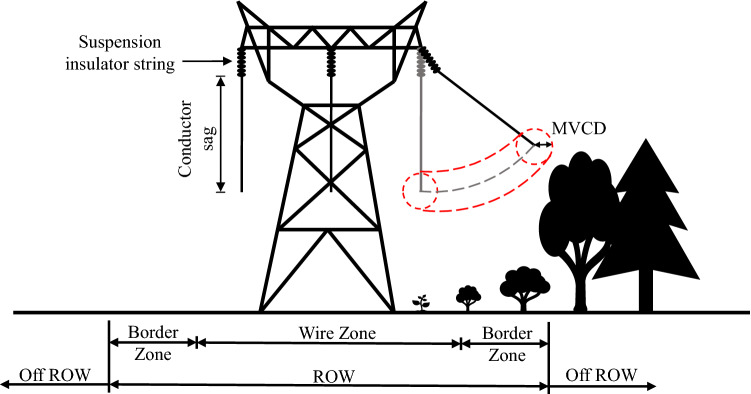
Transmission line right-of-way.

In order to prevent the electric power infrastructure from vegetation interruptions, clearance regulations are universally established. Where powerline-related wildfire risk is concerned, stricter regulations can be introduced. In the United States, the NERC FAC 003-4 standard is the most relevant to transmission system vegetation management^21^. In essence, it requires that a minimum vegetation clearance distance (MVCD) be maintained between transmission conductors and contiguous vegetation. The “wire-border zone” is an effective technique in transmission system vegetation management and is widely used in the field^33^. This approach establishes a right-of-way (ROW) along transmission facilities, as shown in Fig. 3. Typically, the ROW is composed of a wire zone where only low-growing vegetation is allowed and two border zones where taller shrubs and small trees may be permissible. Considering the conductor sag and sway, the width of the ROW is usually much larger than what is needed for structural placement only. For example, the ROW of 230 kV lines can vary between 20 m and 60 m. Note that in Fig. 3 vegetation and conductor movement are only drawn on one side and the MVCD is indicated as a radius surrounding the conductor. Since the position of a conductor is constantly changing due to various loading, a potential flashover zone can be identified along the trajectory.

## Limit state

As mentioned above, the failure scenario under investigation is that the conductor sways outward and becomes close enough to the vegetation to potentially cause flashover and ignite a fire. The air gap between the conductor and the vegetation can be regarded as an insulator whose insulation capacity depends on its size and the ambient characteristics (e.g., temperature, humidity). In terms of the gap size, there are two main sources of uncertainty: one is the turbulent wind loading which directly influences the conductor and vegetation motion; the other is the vegetation growth which is affected by natural conditions and human interventions (e.g., periodic trimming). Vegetation growth is meaningful only over long time horizons (months, years), and its effect can be neglected in the context of short-duration strong wind events. Therefore, the gap size is primarily affected by wind-induced conductor displacement, as vegetation movement is usually deemed negligibly small in comparison. This study defines the failure state (i.e., limit state) as the encroachment of the conductor into the MVCD. In the framework of wildfire risk analysis, it is important to recognize that reaching this limit state is only the first step in the encroachment-flashover-ignition chain of events and the conditional probabilities of occurrence of the other two steps should be considered to compute the overall risk of ignition.

To accurately calculate the flashover probability over an air gap that changes in size during the wind event, the relationship between the gap size and its insulation capacity should be quantified. For example, the Gallet equation was adopted in the NERC FAC 003-4 standard to compute a MVCD yielding a flashover probability of $10^{-6}$ or less^21^. However, further experiments are needed for validation and for better understanding of the transient flashover phenomenon^31,34^. The probability of ignition from flashover varies with many factors, including the flammability of the vegetation and the air conditions near the incident. Given the limited knowledge and significant uncertainties involved, the transformation from MVCD encroachment to ignition relies on subjective judgment and risk attitude of the decision makers. For this reason, the calculation of the aforementioned conditional probabilities is beyond the scope of this work, which instead focuses on the encroachment itself.

The next section presents a mathematical expression of the limit state as well as the methodology for determining the needed quantities.

## Methodology

### Finite element model of a transmission line section

**Figure 4:**
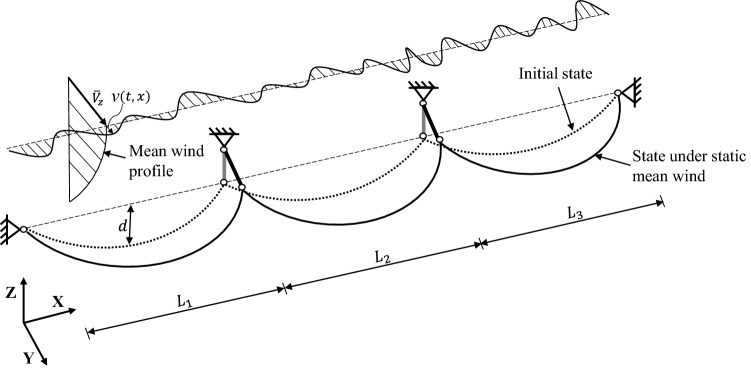
Multi-span transmission line section subject to turbulent wind.

Transmission lines are typically designed in sections, where a TL section consists of multiple spans and can run for up to several kilometers. Fig. 4 illustrates the model of an example multi-span TL section. Herein, the OpenSeesPy environment is used to describe how a finite element model of a multi-span transmission cable can be built and analyzed^35^. The two ends are connected to strain towers allowing no longitudinal conductor movement, and they are modeled as hinged supports. Suspension insulator strings hung at intermediate transmission towers support conductors at their lower ends. The conductor-insulator attachment point is modeled as a hinge, according to the most commonly used articulated suspension clamp^36^. As the insulator string swings, the attachment point can move freely in space. Depending on the voltage, a single conductor (up to 220 kV) or bundled conductors (220 kV and above) can be used in transmission circuits. A single conductor can be modeled using the cable element^37^, while a model of bundled conductors may require the effect of spacers be captured. The conductor takes a catenary form within one span, and the unstrained profile needs to be determined first in order to compute the sagged profile^14^. Suspension insulator strings are usually made of brittle materials (e.g., glass, porcelain), and their bending stiffness is negligibly small. Thus, the suspension insulator string was modeled by the corotational truss element with high axial rigidity, taking account of its large displacements. The length of the insulator string (several meters) varies depending on the voltage, so the wind load applied directly on them is negligible compared to the wind load from the conductor. The specific mechanical parameters needed to set up the finite element model are provided in the Application section by virtue of a two-span transmission line example.

### Description of turbulent wind and buffeting wind load

Although the TL-vegetation interaction is a localized problem, the mathematical wind flow models should be established at large scale for the considered synoptic (non-tropical) wind storms. The wind flow is considered horizontally homogeneous, as transmission systems mostly spread across open terrain areas which provide sufficiently long fetch. However, it should be recognized that non-homogeneity exists when the system encounters trees sporadically (sparse woods or dense forests). While this paper is aimed at proposing a general purpose methodology, separate studies should be conducted for specific conditions to obtain results tailored to those cases. As shown in Fig. 4, it is assumed that the wind flow is present in one direction only, i.e., perpendicular to the span direction of the TL section. This direction is chosen because it is considered the most unfavorable for conductor displacement responses. This assumption leads to a conservative estimation of the risk. To determine the degree of this overestimation, a specific analysis on wind patterns and prevalent wind directions should be conducted for the investigated region. In wind engineering, the total fluctuating wind velocity is usually split into two parts: the constant mean wind velocity $\overline{V}_z$ at height *z*, plus the zero-mean turbulent fluctuation *v*(*t*, *x*), where *t* indicates time and *x* is the location along the conductor cable. Within the lower layer of the atmospheric boundary layer, the variation of mean wind speed with height can be described by the logarithmic law:

1 $$\begin{aligned} \overline{V}_z = \frac{1}{k} \cdot u_* \cdot \textrm{ln}\left( \frac{z}{z^{}_0}\right) \; \end{aligned}$$

where $u_*$ is the shear velocity of the wind flow; $z^{}_0$ is the surface roughness; and *k* is Von Karman’s constant and is usually taken as 0.4.

The 10-min wind speed measured at 10 m above the ground – standard height for mounting anemometers – is typically chosen as the reference wind speed for the mean wind profile. In this study, the intensity of the wind event is described in terms of the reference wind speed (denoted by $\overline{V}_{10}$) from which mean wind speeds at other heights are calculated. For cases where measurements from different averaging times are preferred, conversion factors can be found in the literature^38,39^.

In the initial state, the conductor is typically sagged with a sag-to-span ratio of 1/50~1/30^40^. The mean wind speed along one span can be well approximated by the mean wind speed at the reference height, which is (2/3)*d* below the support level, where *d* is the sag at mid-span^41^. The wind turbulence is correlated in time and space. Both correlations have been studied extensively and well established models for them are available in the literature. As expected, the correlation within the wind field decays with increasing time lag and space separation. At a single point in space, the temporal correlation of alongwind turbulence is most commonly described by the following single-sided power spectral density (PSD) in the frequency domain^42,43^:

2 $$\begin{aligned} \frac{fS_v(f)}{u_{*}^2} = \frac{200fz/\overline{V}_z}{{(1+50fz/\overline{V}_z)}^{5/3}}\; \end{aligned}$$

where *f* is the frequency in Hz. The spatial correlation between the wind velocity fluctuation at two points at the same height (e.g., the reference height) can be captured by the coherence function proposed by Davernport^44^:

3 $$\begin{aligned} \gamma (x^{}_1, x^{}_2, f) = \textrm{exp}\left( -\frac{C|x^{}_1-x^{}_2|f}{\overline{V}_z}\right) \; \end{aligned}$$

where $x^{}_1$ and $x^{}_2$ are the longitudinal coordinates of two points along the TL; *C* is the decay factor and can be set to 16 for horizontal separation. Even though there are different models in the literature^45^, in this paper Gaussianity is assumed for the wind flow velocity fluctuations, based on the work by Einar N. Strømmen^46^. In conclusion, the wind fluctuation component *v*(*t*, *x*) is characterized as a zero-mean, stationary, Gaussian, one dimensional (1D), and multi-variate (mV) random process.

**Figure 5:**
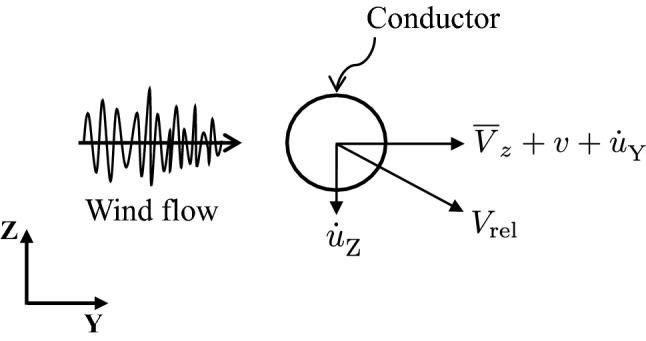
Conductor-wind relative motion.

The buffeting wind load on the conductor is generated by two sources: the total fluctuating wind flow, and the conductor-wind interaction due to conductor motion. Adopting the quasi-steady assumption, the dynamic wind drag force is calculated using Eq. (4) so that also aerodynamic damping is (indirectly) considered:

4 $$\begin{aligned} f^{}_{\textrm{D}} = \frac{1}{2}\rho D C_{\textrm{d}} V_{\textrm{rel}}^{2}\; \end{aligned}$$

where $f^{}_{\textrm{D}}$ is the drag force per unit length; $\rho$ is the air density; *D* is the diameter of the conductor; $C_{\textrm{d}}$ is the drag coefficient; $V_{\textrm{rel}}$ is the relative velocity between conductor and wind flow (see Fig. 5) and is given by the following equation:

5 $$\begin{aligned} V_{\textrm{rel}} = \sqrt{(- \dot{u}^{}_{\textrm{Z}})^2 + (\overline{V}_z + v - \dot{u}^{}_{\textrm{Y}})^2}\; \end{aligned}$$

where $\dot{u}^{}_{\textrm{Z}}$ and $\dot{u}^{}_{\textrm{Y}}$ are the conductor velocities in the Z direction and Y direction, respectively.

### Wind-induced stochastic dynamic response by spectral analysis

The wind-induced buffeting response of a TL section can be computed in two steps^40^: first, the equilibrium state of the structure under gravity and mean wind load is determined by static analysis; second, the dynamic response due to the fluctuating wind component is obtained with the structure linearized at the mean wind state. Ma et al.^14^ validated the linearization of the structure under significant mean wind load . With the two linearizations – the linear relationship between wind velocity and wind load (small fluctuating component assumption), and the linear behavior of the structure characterized at the mean wind state – the properties of the wind fluctuation component (Gaussian, stationary, etc.) will hold for the displacement response as well^46^. To study the probability of encroachment into MVCD, the main task is to obtain the probabilistic properties of the conductor displacement response, i.e., mean and standard deviation in this Gaussian case. Thus, the modal frequency domain approach was used in the second step. Standard deviations were directly derived from the cross-spectral density matrix of the response, which can be found through efficient frequency domain analysis. Note that neither wind field simulation nor expensive Monte Carlo simulation in the time domain is needed.

Following the frequency domain analysis approach, the dynamic response around the mean wind state is separated into background response and resonant response. Mode shapes and modal frequencies of the linearized structure can be obtained by eigenvalue analysis. Then, the cross-spectral density matrix (CSDM) of the modal displacement vector is determined by:

6 $$\begin{aligned}{{\varvec{S}}}_{\textrm{q}}(f) = {{\varvec{H}}}(f) {{\varvec{S}}}_{\textrm{p}}(f) [{{\varvec{H}}}^*(f)]^{\textrm{T}}\; \end{aligned}$$

7 $$\begin{aligned}&{{\varvec{H}}}(f) = [{{\varvec{K}}} + \textrm{i} (2 {\pi } f)({{\varvec{C}}} + {{\varvec{C}}}_{\textrm{aero}}) - (2 {\pi } f)^2 {{\varvec{M}}}]^{-1}\; \end{aligned}$$

where $\varvec{H}(f)$ is the transfer matrix and is expressed in Eq. (7); superscripts * and $\textrm{T}$ represent complex conjugate operator and transpose operator, respectively; $\textrm{i} = \sqrt{-1}$; $\varvec{K}$, $\varvec{C}$, $\varvec{C}_{\textrm{aero}}$, and $\varvec{M}$ are generalized stiffness matrix, generalized structural damping matrix, generalized aerodynamic damping matrix, and generalized mass matrix in the modal space, respectively^47^. It is worth pointing out that $\varvec{C}_{\textrm{aero}}$ is non-diagonal because of the coupling effects among mode shapes. Additionally, $\varvec{S}_{\textrm{p}}(f)$ is the CSDM of the modal load vector and can be calculated as:

8 $$\begin{aligned}&{{\varvec{S}}}_{\textrm{p},jk} = \frac{4 \bar{f}_{\textrm{D}}^2 {{\varvec{S}}}_v(f)}{\overline{V}_z^2} |{{\varvec{J}}}_{jk}(f)|^2\; \end{aligned}$$

9 $$\begin{aligned}&|{{\varvec{J}}}_{jk}(f)|^2 = \int _0^L \int _0^L \gamma (x^{}_1, x^{}_2, f) {{\varvec{\varphi }}}^{}_{\textrm{Y}j}(x^{}_1) {{\varvec{\varphi }}}^{}_{\textrm{Y}k}(x^{}_2) \textrm{d}x^{}_1 \textrm{d}x^{}_2\; \end{aligned}$$

where $\bar{f}^{}_{\textrm{D}}$ is the static mean drag force per unit length with $\overline{V}_z = V_{\textrm{rel}}$ in Eq. (4); $|\varvec{J}_{jk}(f)|^2$ is the joint acceptance function; *L* is the total span length of the TL section; $\varvec{\varphi }^{}_{\textrm{Y}j}(x^{}_1)$ is the Y component at $x^{}_1$ in the *j*-th mode; $\varvec{\varphi }^{}_{\textrm{Y}k}(x^{}_2)$ is the Y component at $x^{}_2$ in the *k*-th mode ($x_1$ and $x_2$ are just integration variables). Notice that only Y components appear in Eq. (9), because the wind flow is in the Y direction only.

Once $\varvec{S}_{\textrm{q}}(f)$ is obtained from Eq. (6), the standard deviation of the total displacement response at the *r*-th node is derived by integration over the frequency range^47^:

10 $$\begin{aligned} \sigma ^{}_{\lambda r} = \sqrt{\int _0^{\infty } \sum _{j=1}^{N} \sum _{k=1}^{N} {{\varvec{\varphi }}}^{}_{\lambda j r} {{\varvec{\varphi }}}^{}_{\lambda k r} {{\varvec{S}}}_{\textrm{q},jk} (f) \textrm{d}f}\; \end{aligned}$$

where *N* is the total number of modes considered; $\lambda \in \left\{ \text {X, Y, Z} \right\}$ indicates the direction.

The background response is considered quasi-static and its standard deviation, $\sigma ^{}_{\lambda r, \textrm{B}}$, can be calculated as described above, but computing the transfer function simply as $\varvec{H}(f) = \varvec{K}^{-1}$, instead of using Eq. (7). Finally, the standard deviation of the resonant response, $\sigma ^{}_{\lambda r, \textrm{R}}$, is calculated as follows:

11 $$\begin{aligned} \sigma ^{}_{\lambda r, \textrm{R}} = \sqrt{\sigma _{\lambda r}^2 - \sigma _{\lambda r, \textrm{B}}^2}\; \end{aligned}$$

### Mathematical description of real-time vegetation clearance

**Figure 6:**
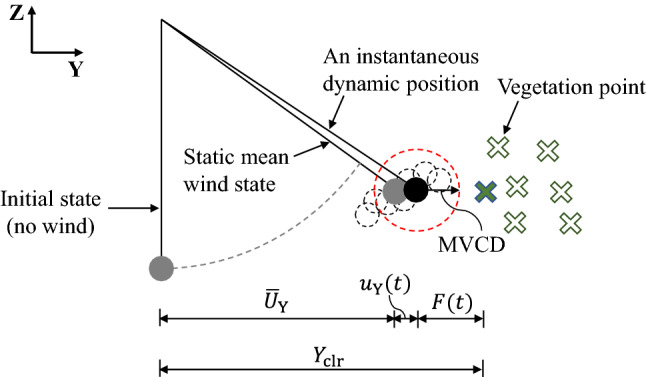
Cross sectional view of vegetation clearance.

As previously mentioned, the determination of the limit state involves two factors, i.e., conductor displacement and vegetation clearance. In terms of conductor displacements, the effects of insulator swing are included in the buffeting response of the conductor and are captured by its probabilistic properties. When the wind flow is in the Y direction only, the displacement response in the longitudinal direction (X) is considerably smaller than that in the alongwind direction (Y) or crosswind direction (Z). This study is concerned with the lateral (Y-direction) vegetation clearance, and a simplified configuration is illustrated in Fig. 6. For simplicity, the conductor swinging is only drawn on one side, with wind blowing in the positive Y direction.

During a high wind event, the real-time clearance is only affected by conductor movement as both vegetation growth and vegetation motion are neglected. The conductor position dynamically changes in the space around the mean wind state (indicated by dashed circles) with a radial MVCD zone moving with it. The vegetation (tree) nearby is represented by vegetation points for which data from the latest survey may be used (e.g., point cloud data from a LiDAR survey). It should be recognized that in reality vegetation has great diversity and complexity (e.g., shape, species) which are not captured by vegetation points. The mathematical expression of the real-time lateral clearance can be written as:

12 $$\begin{aligned} F(t) = Y_{\textrm{clr}} - \overline{U}^{}_{\textrm{Y}} - u^{}_{\textrm{Y}}(t)\; \end{aligned}$$

where *t* is the time instant; $Y_{\textrm{clr}}$ is the known pre-event clearance measured laterally from the cable resting state to the nearest vegetation point (indicated as solid cross); $\overline{U}^{}_{\textrm{Y}}$ and $u^{}_{\textrm{Y}}(t)$ are static mean displacement and dynamic displacement in the Y direction, respectively. The violation of MVCD (limit state) occurs when $F(t) < mvcd$, where *mvcd* is a prescribed value that can be determined based on voltage, altitude, etc^21^.

**Figure 7:**
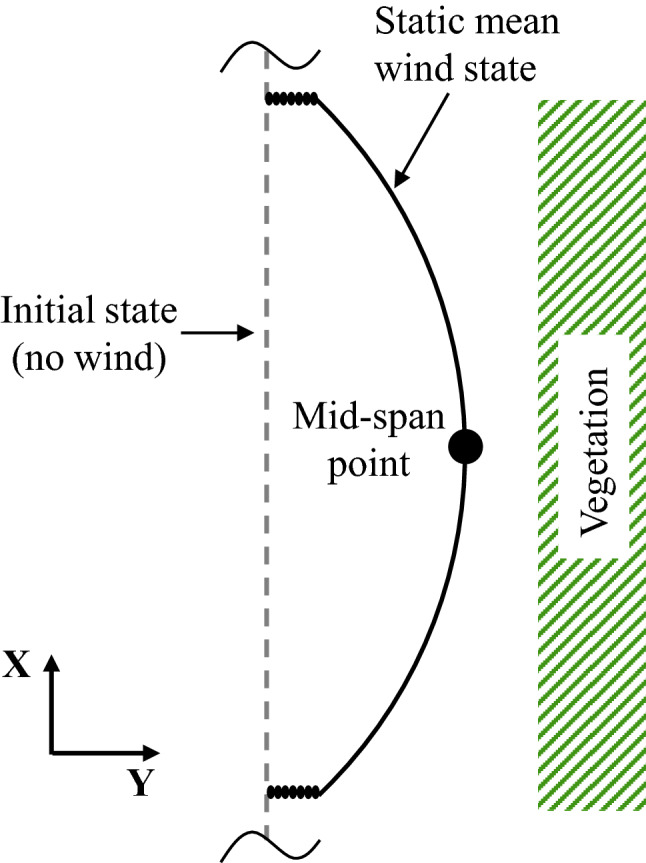
Top view of the lateral clearance within one span.

At this point two additional concepts need to be made clear. First, Eq. (12) is meaningful on the underlying premise that the displacement of the conductor under mean wind establishes the possibility of violating MVCD under dynamic wind loads, and yet dynamic response effects are worth considering, as shown in Fig. 6. For the case where the displaced conductor is too far from the vegetation ($Y_{\textrm{clr}} \gg \overline{U}^{}_{\textrm{Y}}+mvcd$), violation can be deemed impossible; whereas if the conductor under mean wind is already too close to the vegetation ($Y_{\textrm{clr}} \le \overline{U}^{}_{\textrm{Y}}+mvcd$), no calculation is needed since violation is a certain event. This situation is actually relatively common for vegetation clearance designed for ordinary wind loads. Second, the blown-out envelope within one span is influenced by the changing sag of the conductor. The maximum lateral displacement within a span is achieved at the mid-span in coincidence with the maximum sag, as shown in Fig. 7. Moreover, if constant vegetation configuration is enforced for an entire span, the mid-span point will stand critical for limit state check.

### Probability of first-excursion failure

The violation of MVCD, as a proxy for utility-induced ignition, could cause large-scale blackout and disastrous wildfires the very first time it occurs. This type of failure is categorized as failure due to first excursion (up-crossing), a problem extensively investigated by random vibration theory^48^. As previously mentioned, the fluctuating displacement $u^{}_{\textrm{Y}}(t)$ can be characterized as a stationary, Gaussian, and zero-mean random process. Letting $F(t)=mvcd$ and rearranging Eq. (12), the up-crossing threshold *a* is expressed as:

13 $$\begin{aligned} a = Y_{\textrm{clr}} - \overline{U}^{}_{\textrm{Y}} - mvcd\; \end{aligned}$$

Note that Eqs. (12) and (13) are formulated in a continuous sense, however, calculations are actually performed at nodes of the finite element model. Thus, the expected excursion rate (i.e., the average number of up-crossings per unit time) at the *r*-th node with respect to threshold $a_{r}$ can be calculated as^49^:

14 $$\begin{aligned} v_{ar}^{+} = \frac{1}{2 \pi } \frac{\sigma ^{}_{\dot{\textrm{Y}}r}}{\sigma ^{}_{\textrm{Y}r}} \textrm{exp}(-\frac{a_{r}^2}{2 \sigma _{\textrm{Y}r}^2})\; \end{aligned}$$

where $\sigma ^{}_{\textrm{Y}r}$ can be obtained using Eq. (10) with $\lambda =$ Y; $\sigma ^{}_{\dot{\textrm{Y}}r}$ is the standard deviation of Y-direction velocity response at the *r*-th node and can be computed by:

15 $$\begin{aligned} \sigma ^{}_{\dot{\textrm{Y}} r} = \sqrt{\int _0^{\infty } \sum _{j=1}^{N} \sum _{k=1}^{N} {{\varvec{\varphi }}}^{}_{\textrm{Y} j r} {{\varvec{\varphi }}}^{}_{\textrm{Y} k r} (2\pi f)^2 {{\varvec{S}}}_{\textrm{q},jk} (f) \textrm{d}f}\; \end{aligned}$$

Furthermore, it is found that significant aerodynamic damping renders the background response dominant in the dynamic response^47^. This indicates that $u^{}_{\textrm{Y}}(t)$ is far from a narrow-banded process, which would instead require dominance of the resonant response. Thus, with a further assumption that excursions arrive independently in the time domain, the probability of encroachment, formulated as the probability of up-crossing excursion ($u^{}_{\textrm{Y}}(t) > a$) in the interval $0<t<T^{}_{0}$, is given by^49^:

16 $$\begin{aligned} P_{\textrm{en},r}(T^{}_{0}) = 1 - \textrm{exp}(-v_{ar}^{+} T^{}_{0})\; \end{aligned}$$

where $T^{}_{0}$ is the time horizon or duration in seconds. One particular advantage of computing the probability $P_{\textrm{en},r}(T^{}_{0})$ is that it takes into account the effect of $T^{}_{0}$. In practical applications, $T^{}_{0}$ is not necessarily equal to the forecast wind event duration but can be any shorter duration of interest. In general terms, the longer the waiting time, the more likely excursion will occur. This can be very helpful in time-sensitive decision making where risks evolve with time.

## Application

While the proposed methodology is general, and can be applied to power transmission systems with different characteristics and in different regions, two specific application examples are presented, to demonstrate the approach. The methodology was first implemented at the single TL section level, and then results were extended to illustrate the application at the system level.

### Example of a two-span transmission line section

**Figure 8:**
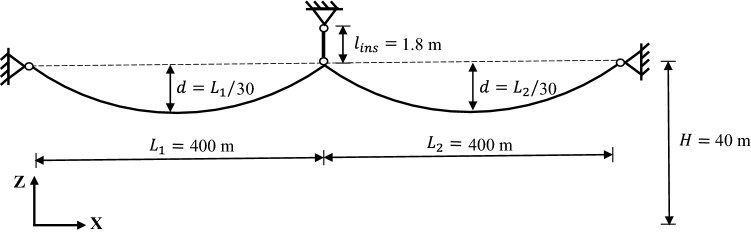
Sketch of the two-span TL section model (not to scale).

A two-span TL section with nominal voltage of 230 kV (alternating current) was first studied, as shown in Fig. 8. General information on the element type and computational environment was already provided in the Methodology section. For this particular example, relevant modelling details are given as following. The conductor is hung at all towers at the same height ($H = 40$ m) with the largest sag at mid-span $d = 13.33$ m. The conductor is of the “Drake” type and relevant properties are: diameter $D = 0.028$ m, unit weight $w = 15.966$ N/m, modulus of elasticity $E = 77$ GPa. The suspension insulator string was modeled by one co-rotational truss element with the following properties: length $l_{\textrm{ins}} = 1.8$ m, diameter $D_{\textrm{ins}} = 0.254$ m, total mass of the insulator string $M_{\textrm{ins}} = 48$ kg, and modulus of elasticity $E_{\textrm{ins}} = 210$ GPa. In order to account for possible future high wind events, seven intensity levels were studied: $\overline{V}_{10} \in \left\{ 30, 35, 40, 45, 50, 55, 60 \right\}$ m/s. The following parameters are needed too: surface roughness $z^{}_0 = 0.03$ m (open terrain), drag coefficient $C_{\textrm{d}} = 1.0$, air density $\rho = 1.226$ kg/$\textrm{m}^3$, and gravitational acceleration $g = 9.81$ m/$\textrm{s}^2$. Note that thermal loading or any other physical loading (e.g., ice) was neglected in this example.

It is taken for granted that vegetation data is known beforehand, either from previous survey or valid estimation. The *mvcd* value corresponding to 230 kV voltage varies between 1.2 m and 1.6 m depending on the altitude^21^. It was assumed that a constant $mvcd = 1.4$ m is required throughout the entire TL section. Critical checking points can be identified based on available knowledge of TL and pertaining vegetation. For this example analysis, the vegetation clearance was assumed constant along the TL section, and logically the mid-span point of either span was chosen as the checking point. As mentioned earlier, for Eq. (12) to be meaningful, $Y_{\textrm{clr}} > \overline{U}^{}_{\textrm{Y}}+mvcd$ should be satisfied at any location to be checked. Correspondingly, a wide range of $Y_{\textrm{clr}}$ values with 0.5 m interval were selected for analysis 18.0, 18.5, ..., 26.5, 27.0 m.

First, static structural analysis under mean wind load was carried out for each considered wind intensity, and the results are summarized in Table 1. Owing to the symmetry of both structure and load, the two mid-span points experience the same displacements while the conductor-insulator attachment point has no longitudinal displacement. The conductor mid-span exhibits noticeable displacements in the alongwind direction and crosswind direction, and both increase as wind intensifies. This is mainly due to the rigid-body swing (considering the large sag) and partly due to the elongation of the conductor. With the wind load on the insulator string neglected and the relatively small weight thereof, the insulator string swings out due to the drag force from the connected conductor, and it is found that the insulator sway angle $\bar{\theta }_{ins}$ is consistent with the sway angle of the conductor plane. Moreover, the rate of increase of the sway angle is reduced as the TL approaches positions almost parallel to the wind flow. It can be shown from simple calculations $\left(\sqrt{\overline{U}_{\textrm{Y,att}}^2 + (l_{\textrm{ins}} - \overline{U}^{}_{\textrm{Z,att}})^2}\right)$ that the length of the insulator string does not change in any significant way thanks to its high rigidity. In comparison with the magnitude of mid-span displacements and *mvcd*, the influence of the insulator string sway has non-negligible contribution to the overall displacements of the conductor.

**Table 1:** Displacements under mean wind load.

$\overline{V}_{10}$	$\overline{V}_{\textrm{ref}^{\textrm{a}}}$	At mid-span point (m)			At attachment point (m)			$\bar{\theta }_{\textrm{ins}^{\textrm{b}}}$
(m/s)	(m/s)	$\overline{U}_{\textrm{X,mid}}$	$\overline{U}_{\textrm{Y,mid}}$	$\overline{U}_{\textrm{Z,mid}}$	$\overline{U}_{\textrm{X,att}}$	$\overline{U}_{\textrm{Y,att}}$	$\overline{U}_{\textrm{Z,att}}$	(degree)
30	35.9	$-0.026$	12.081	5.480	0.000	1.431	0.708	52.7
35	41.8	$-0.034$	13.499	7.045	0.000	1.566	0.912	60.5
40	47.8	$-0.041$	14.560	8.295	0.000	1.648	1.076	66.3
45	53.8	$-0.047$	15.413	9.265	0.000	1.698	1.202	70.6
50	59.8	$-0.053$	16.147	10.016	0.000	1.729	1.299	73.8
55	65.7	$-0.058$	16.814	10.603	0.000	1.749	1.374	76.3
60	71.7	$-0.062$	17.441	11.068	0.000	1.762	1.434	78.3

$^{\textrm{a}}$Mean wind velocity at the reference height ($\approx {31.1}$ m).

$^{\textrm{b}}$Sway angle of the suspension insulator string, $\bar{\theta }_{\textrm{ins}} = \textrm{arctan}[\overline{U}^{}_{\textrm{Y,att}}/(l_{\textrm{ins}} - \overline{U}_{\textrm{Z,att}})]$.

**Figure 9:**
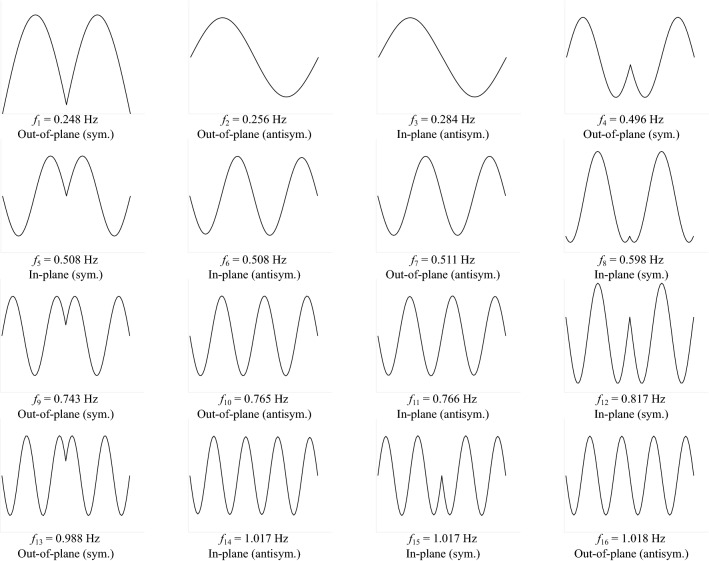
Modal frequencies and mode shapes ($\overline{V}_{10} = 45$ m/s).

With the structural behavior linearized at the mean wind state, the dynamic modal properties of the linear system were obtained from eigenvalue analysis in the displaced state. It is customary to describe the conductor movement (like a pendulum) using in-plane and out-of-plane modes. For instance, Fig. 9 displays the first 16 modal frequencies and mode shapes corresponding to $\overline{V}_{10} = 45$ m/s. Note that the mode shapes are either symmetric (sym.) or antisymmetric (antisym.). Pairs of in-plane and out-of-plane modes that share similar shapes and frequencies can be observed, such as modes 2 and 3, modes 4 and 5, etc. Significant coupling effects of these pairs will lead to non-zero off-diagonal terms in $\varvec{C}_{\textrm{aero}}$^47^.

**Figure 10:**
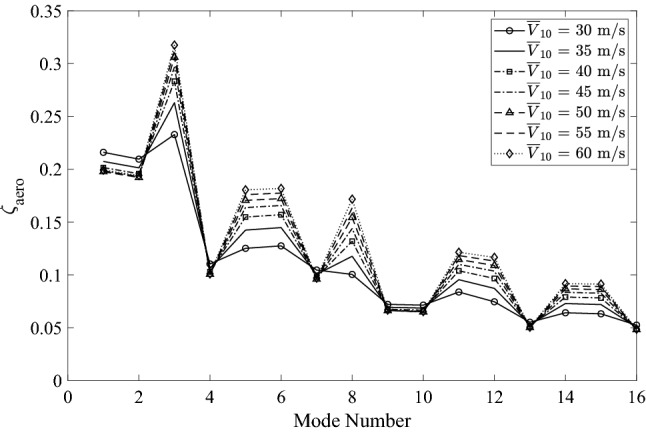
Comparison of aerodynamic damping ratios.

The dynamic response around the static deflected position was computed in the frequency domain using the first 16 modes. This number was found to result in sufficient accuracy by a convergence test in terms of standard deviation of the displacement response. Structural damping was neglected as it is very small compared to the dominant aerodynamic damping. The aerodynamic damping ratio of the *j*-th mode can be obtained by:

17 $$\begin{aligned} \zeta _{\textrm{aero},j} = \frac{{{\varvec{C}}}_{\textrm{aero},jj}}{4 {\pi } f_{j} {{\varvec{M}}}_{j}} \end{aligned}$$

where $\varvec{C}_{\textrm{aero},jj}$ corresponds to the *j*-th diagonal term of $\varvec{C}_{\textrm{aero}}$; $f_{j}$ and $\varvec{M}_{j}$ are the modal frequency and the generalized mass of the *j*-th mode, respectively. Fig. 10 compares the modal aerodynamic damping ratios under different wind intensities. It shows that significant aerodynamic damping is present and overall decreases with increasing mode number. Referencing Fig. 9, it can be observed that in-plane modes bear higher aerodynamic damping than out-of-plane modes. Consistent with findings by Stengel et al.^50^, a nonlinear relationship exists between aerodynamic damping ratios and high wind velocities.

**Figure 11:**
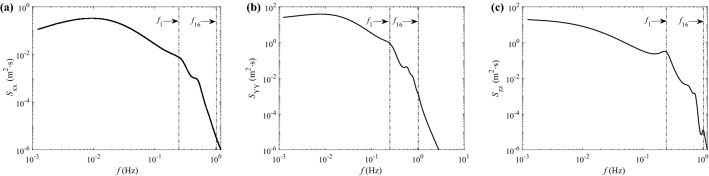
PSDs of displacement response components at mid-span point ($\overline{V}_{10}$ = 45 m/s): (**a**) longitudinal displacement; (**b**) alongwind displacement; (**c**) crosswind displacement.

Fig. 11 gives the power spectral densities of the displacement response components at mid-span, where $f_{\textrm{1}}$ and $f_{\textrm{16}}$ correspond to values in Fig. 9. It is evident that the magnitude of longitudinal displacement is much smaller than the other two. Traces of resonances can be observed in all three directions within the range $f_{\textrm{1}} \le f \le f_{\textrm{16}}$. However, most energy is attributed to the background response (low frequency part) because the resonant response is damped out by the high aerodynamic damping.

**Figure 12:**
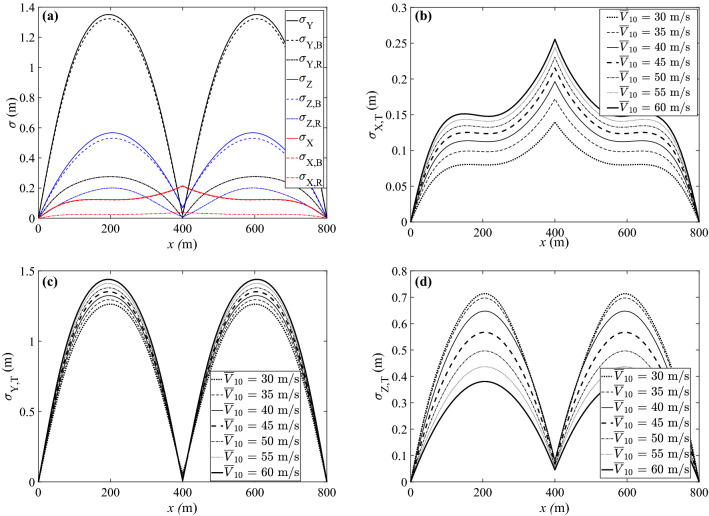
Comparison of standard deviations of displacement responses: (**a**) $\overline{V}_{10}$ = 45 m/s; (**b**) longitudinal displacement; (**c**) alongwind displacement; (**d**) crosswind displacement.

Subsequently, referring to Eqs. (10) and (11), the standard deviations of the displacement response components were obtained for each considered wind intensity. Fig. 12a shows the case with $\overline{V}_{10}$ = 45 m/s; results for other intensity levels are similar. It can be observed that the standard deviation of the background response is dominant in all three directions. Overall, the alongwind displacement shows the highest standard deviation, the crosswind displacement second, and the longitudinal displacement comes the lowest. Moreover, the standard deviations of alongwind and crosswind displacements are symmetric about the attachment point, with maxima appearing at the mid-span. The standard deviation of longitudinal displacement, is also symmetric, but it achieves its maximum at the attachment point. This indicates that the dynamic behavior of the insulator string is more meaningful in longitudinal displacement, which affects the lateral clearance indirectly by shifting the conductor (and in turn the critical checking points) along the span direction.

Focusing on the total standard deviation, Figs. 12b–d examine the variation of the standard deviation with the wind intensity. Mid-span results are given in Table 2, where $\overline{U}^{}_{\textrm{Y,mid}}$ is the same as in Table 1 and is repeated here for convenience; $\delta ^{}_{\textrm{Y}}$ is the coefficient of variation (c.o.v.) of the alongwind displacement and can be calculated by:

18 $$\begin{aligned} \delta ^{}_{\textrm{Y}} = \frac{\sigma ^{}_{\textrm{Y}}}{\overline{U}^{}_{\textrm{Y}}} \end{aligned}$$

**Table 2:** Standard deviations of mid-span displacement responses.

$\overline{V}_{10}$	$\sigma ^{}_{\textrm{X}}$	$\sigma ^{}_{\textrm{Y}}$	$\sigma ^{}_{\textrm{Z}}$	$\overline{U}^{}_{\textrm{Y,mid}}$	$\delta ^{}_{\textrm{Y}}$
(m/s)	(m)	(m)	(m)	(m)	
30	0.080	1.263	0.713	12.081	$10.46\%$
35	0.098	1.295	0.697	13.499	$9.59\%$
40	0.112	1.324	0.647	14.560	$9.09\%$
45	0.124	1.351	0.567	15.413	$8.77\%$
50	0.133	1.379	0.496	16.147	$8.54\%$
55	0.141	1.410	0.436	16.814	$8.39\%$
60	0.148	1.439	0.380	17.441	$8.25\%$

Clearly, the standard deviations of longitudinal displacement ($\sigma ^{}_{\textrm{X}}$) and alongwind displacement ($\sigma ^{}_{\textrm{Y}}$) both increase with increasing wind velocity. However, considering the lateral clearance of the mid-span point, $\sigma ^{}_{\textrm{X}}$ is much smaller than $\sigma ^{}_{\textrm{Y}}$ ($\sigma ^{}_{\textrm{X}} \approx 10\% \sigma ^{}_{\textrm{Y}}$). Therefore, the effects of longitudinal shifting of the conductor were neglected in this example. In contrast to $\sigma ^{}_{\textrm{X}}$ and $\sigma ^{}_{\textrm{Y}}$, the standard deviation of crosswind displacement ($\sigma ^{}_{\textrm{Z}}$) shows a favorable decreasing trend with increasing wind intensity, as in Fig. 12d. Recall that the total standard deviation is dominated by the quasi-static background response which is closely related to the static mean wind position. It is easy to understand that as the static conductor plane becomes more in-plane with the Y-direction fluctuations, less responses will be excited in Z direction. According to Table 2, $\sigma ^{}_{\textrm{Y}}$ values can be very close to or even larger than *mvcd* ($=1.4$ m). The non-dimensional measure c.o.v. shows that the degree of variability of the alongwind displacement at mid-span decreases with the wind intensity increasing, as the increase in $\sigma ^{}_{\textrm{Y}}$ is slower than $\overline{U}^{}_{\textrm{Y,mid}}$. Nevertheless, high variation is anticipated in the conductor displacement response during strong wind events. This constitutes a major finding, because it manifests the necessity of considering in regular vegetation management and risk analysis the wind turbulence-induced dynamic effects and associated uncertainties.

Since the fluctuating displacements at both mid-span points have the same probabilistic properties, i.e., $u^{}_{\textrm{Y}}(t)\sim \mathscr {N}(0, \sigma ^{}_{\textrm{Y}})$, only the left mid-span is discussed in the following. Based on the vegetation clearance configuration in Eq. (12), the probabilities of encroachment at mid-span (denoted by $P_{\textrm{en}}$ omitting subscript *r*) were computed for different wind velocities and varying clearances. In terms of the time horizon ($T^{}_{0}$), an examination of PSPS post event reports suggests that the duration of high wind events varies from several hours to two days^51^. Therefore, $P_{\textrm{en}}$ values were computed for up to 48 hours. Results for $\overline{V}_{10} = 30$ m/s are presented in Table 3, where $u^{}_{\textrm{Y}}(t) \sim \mathscr {N}(0, 1.263)$. It is shown that with an 18 m lateral clearance at mid-span, there is a $100\%$ probability that MVCD violation will happen within the first 24 hours of the wind event. This is attributed to the fact that $\sigma ^{}_{\textrm{Y}}$ $(=1.263$ m) is comparable to the up-crossing threshold *a* $(=4.519$ m). However, without considering the dynamic effects, a deterministic evaluation based solely on the static mean wind position (i.e., $a=4.519$ m) may lead to the contrary conclusion that encroachment does not occur. As $Y_{\textrm{clr}}$ increases to 22.0 m, $P_{\textrm{en}}$ (48 h) reaches a very low level ($10^{-6}$). Thus, results for $Y_{\textrm{clr}} > 22.0$ m are not shown in the table.

**Table 3:** Probability of encroachment at mid-span under $\overline{V}_{10} = 30$ m/s.

$Y_{\textrm{clr}}$	*a*	$v_{a,\textrm{mid}}^{+}$	$P_{\textrm{en}}$ (24 h)	$P_{\textrm{en}}$ (48 h)
(m)	(m)	(1/s)		
18.0	4.519	$1.11 \times 10^{-4}$	1.00	1.00
18.5	5.019	$2.49 \times 10^{-5}$	$8.83 \times 10^{-1}$	$9.86 \times 10^{-1}$
19.0	5.519	$4.77 \times 10^{-6}$	$3.38 \times 10^{-1}$	$5.61 \times 10^{-1}$
19.5	6.019	$7.83 \times 10^{-7}$	$6.54 \times 10^{-2}$	$1.27 \times 10^{-1}$
20.0	6.519	$1.10 \times 10^{-7}$	$9.44 \times 10^{-3}$	$1.88 \times 10^{-2}$
20.5	7.019	$1.32 \times 10^{-8}$	$1.14 \times 10^{-3}$	$2.27 \times 10^{-3}$
21.0	7.519	$1.35 \times 10^{-9}$	$1.17 \times 10^{-4}$	$2.33 \times 10^{-4}$
21.5	8.019	$1.18 \times 10^{-10}$	$1.02 \times 10^{-5}$	$2.05 \times 10^{-5}$
22.0	8.519	$8.88 \times 10^{-12}$	$7.67 \times 10^{-7}$	$1.53 \times 10^{-6}$

**Figure 13:**
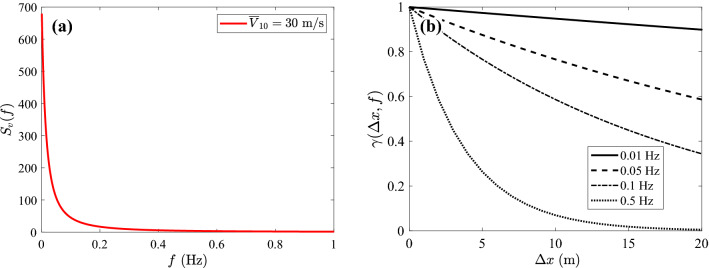
Characterization of turbulent wind with $\overline{V}_{10}$ = 30 m/s: (**a**) PSD from Eq. (2); (**b**) coherence from Eq. (3).

For such a simple case — symmetric structure and regular vegetation clearance — the violation of the MVCD within an entire span can be captured by focusing exclusively on the mid-span point. However, theoretically it is necessary to consider also the likelihood of violation not occurring at mid-span while occurring at near-mid-span locations. The key to address this issue and determine if it is of practical relevance resides in the correlation between the mid-span displacement response and near-mid-span displacement responses. To illustrate this point, the PSD and coherence of the turbulence component (corresponding to $\overline{V}_{10}$ = 30 m/s) are given in Fig. 13. The PSD, shown up to 1 Hz for ease of observation, decreases steeply with the frequency. Fig. 13b examines the frequency-dependent coherence of the wind turbulence with varying spatial distance, $\Delta x = |x^{}_{1}-x^{}_{2}|$. Considering the most relevant frequency range [0, 0.5] Hz, the coherence remains high when the distance between two points is small (e.g., $\Delta x < 2$ m). This implies that the mid-span point, whose dynamic response is highly correlated with nearby points, is anticipated to first violate MVCD given its higher risk profile. Therefore, in the following, the encroachment probability at mid-span is considered to be representative of the entire span and it is expected that a similar approach is viable in most practical situations.

**Figure 14:**
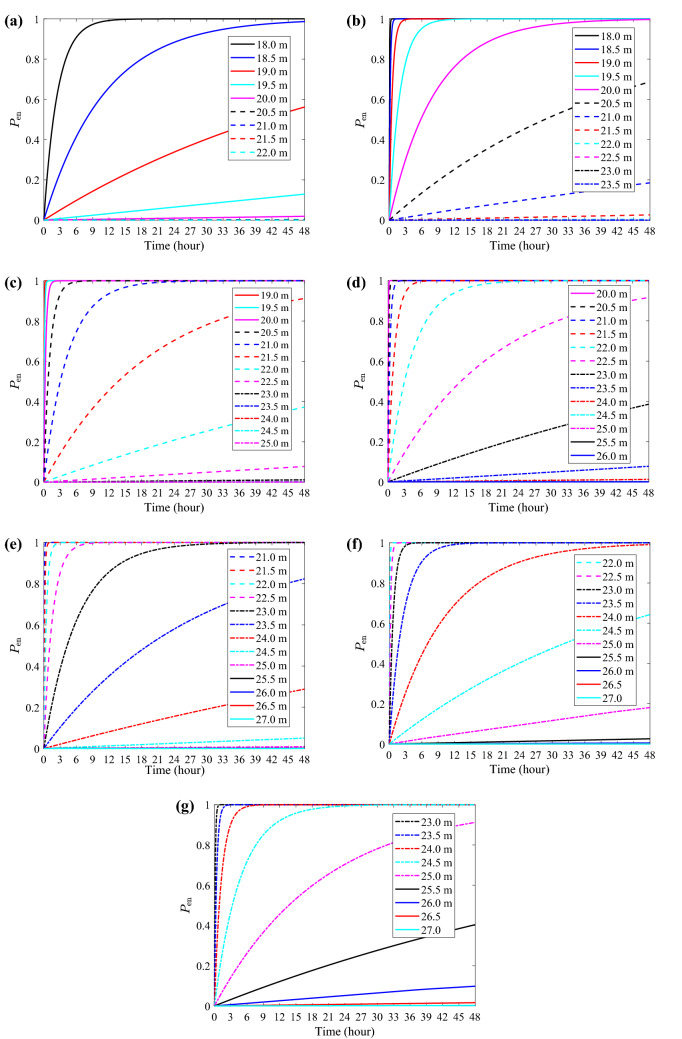
Probability of encroachment with varied clearances: (**a**) $\overline{V}_{10}$ = 30 m/s; (**b**) $\overline{V}_{10}$ = 35 m/s; (**c**) $\overline{V}_{10}$ = 40 m/s; (**d**) $\overline{V}_{10}$ = 45 m/s. (**e**) $\overline{V}_{10}$ = 50 m/s; (**f**) $\overline{V}_{10}$ = 55 m/s; (**g**) $\overline{V}_{10}$ = 60 m/s.

The wind intensity level and the vegetation clearance policy are major factors affecting the probability of encroachment. The sensitivity of the probability of encroachment with respect to these two factors can be best understood from Fig. 14, where the performance of different wind clearance policies considering two-day wind events with different intensities is compared. Note that different $Y_{\textrm{clr}}$ ranges were examined depending on the wind intensity level, as indicated in the legends. It is evident that the $P_{\textrm{en}}$ escalates as the TL continues operating during a wind event. For each considered wind intensity, a narrow $Y_{\textrm{clr}}$ range can be identified within which small changes can have important impact on the probability of encroachment. This clearance range can be a useful reference for cost-effective vegetation management planning. Moreover, the effectiveness of certain clearance options is sensitive to the wind intensity. For instance, the $P_{\textrm{en}}$ (48 h) sustained by a 24.0 m clearance rises from $1.52\times 10^{-4}$ (acceptable) to $1.24\times 10^{-2}$ (alarming) as $\overline{V}_{10}$ increases from 40 m/s to 45 m/s, as in Figs. 14c,d. In the context of decision making towards PSPS, data on vegetation and transmission assets are usually known beforehand, while wind data are available from weather forecast. Then probabilities of encroachment can be calculated across the transmission network for a specified duration, which will help predict potential ignition locations. It should be emphasized again that the de-energization decision is not driven by consideration on an individual span or TL, but is based on system-level analysis considering power flow. The scope of power shutoff is a result of weighing two risks: the risk of catastrophic wildfires caused by utility assets, and the risks and certain drawbacks resulting from leaving the public without electricity.

### Example of a transmission system

**Figure 15:**
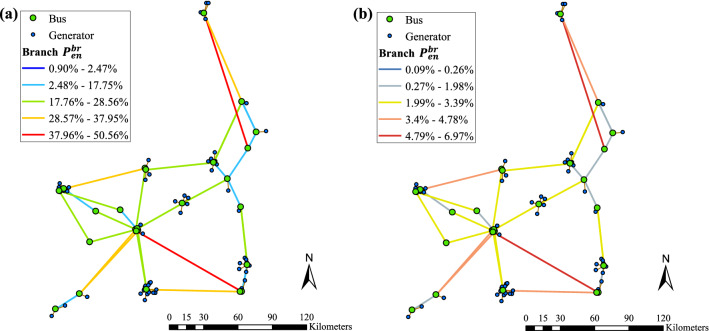
Map of the power transmission system and its probability of encroachment under a 48 h strong wind event: (**a**) $Y_{\textrm{clr}} = 20.5$ m, $P_{\textrm{en}}$ (48 h)$= 2.27\times 10^{-3}$; (**b**) $Y_{\textrm{clr}} = 21.0$ m, $P_{\textrm{en}}$ (48 h) $= 2.33\times 10^{-4}$.

**Figure 16:**
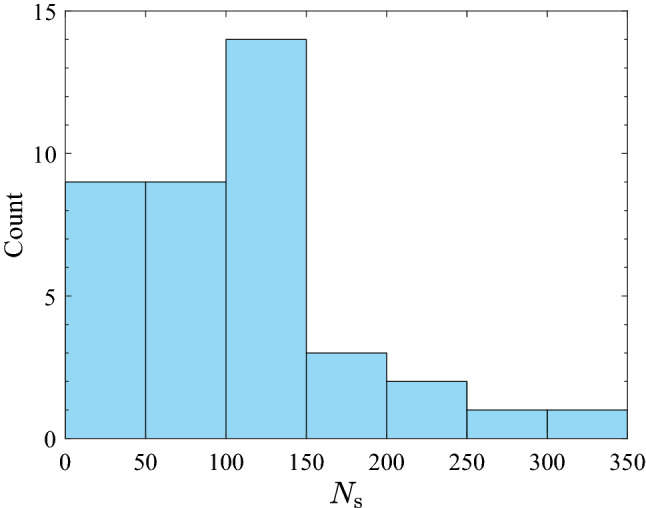
Frequency distribution of $N_{\textrm{s}}$.

A real-world transmission network presents tremendous variations and uncertainties not only in its structural and electrical aspects, but also in the surrounding conditions. Nevertheless, following a procedure similar to the one discussed above, a separate study can be conducted efficiently at any location of interest for which data is supplied. The aims of this system-level example are twofold. First, it serves to illustrate the incorporation of span-wise encroachment probability into the analysis scale at which the de-energization decision actually made. Second, it is used to demonstrate how the branch lengths (in terms of the number of conductor spans) affect the overall probability of encroachment. The transmission system example is based on the benchmark Reliability Test System - Grid Modernization Laboratory Consortium (RTS-GMLC) model^52^, but only region 3 is used which is sized in such a way to realistically represent southern California. The data is publicly available^53^. As shown in Fig. 15, the system consists of 25 buses, 69 generators, and 39 transmission branches connecting buses. However, the RTS-GMLC dataset is lacking in structure-related information for each branch, such as supporting structures, span length, sag, etc. Thus, for illustrative purposes, it was assumed that all transmission branches are composed of the same two-span sections (as shown in Fig. 8) and all related configurations still hold. Considering a three-phase circuit blown out to one side (see Fig. 3), the failure (i.e., encroachment into MVCD) within one span is caused by the mid-span point of the outer-phase conductor. For a transmission branch, encroachment is defined as the event in which any of its spans violates MVCD; therefore a branch can me modeled as a classical series system. It was further assumed that the encroachment failures among different spans are statistically independent, which is justified by the previous considerations on the correlation distance of the wind fluctuations. Hence, the probability of encroachment into MVCD of a transmission branch, $P_{\textrm{en}}^{\textrm{br}}$, is expressed as:

19 $$\begin{aligned} P_{\textrm{en}}^{\textrm{br}} = 1 - (1-P_{\textrm{en}})^{N_{\textrm{s}}} \end{aligned}$$

where $N_{\textrm{s}}$ is the number of spans of the considered branch. In this example, $N_{\textrm{s}}$ was obtained by dividing each branch into 400 m-long spans with rounding off at ends. Depending on the length of branches, $N_{\textrm{s}}$ ranges between 4 and 310, as shown by the histogram in Fig. 16.

With wind intensity and event duration fixed (48 hours), the effectiveness of a certain clearance is preferably examined at the branch or system level. Taking advantage of results in Table 3, two lateral clearances (20.5 m and 21.0 m) under $\overline{V}_{10}=$ 30 m/s are compared in Fig. 15. Although the span-level $P_{\textrm{en}}$ is very low, the branch-level $P_{\textrm{en}}^{\textrm{br}}$ can be considerably high. As expected, longer branches have higher probability of encroachment because of the larger number of spans. This indicates the importance of stricter clearances for longer branches on the premise of “series system failure”. For instance, the $P_{\textrm{en}}^{\textrm{br}}$ of the longest branch can be reduced from $50.56\%$ to $6.97\%$ by increasing clearance from 20.5 m to 21.0 m. Leveraging forecasted weather data, the system-wide encroachment probabilities as visualized in Fig. 15 can help evaluate power shutoff decisions both spatially and temporally. Additionally, the accuracy of encroachment prediction can benefit from improving data quality.

## Conclusions

This paper presents a methodology for assessing the probability of wildfire ignition from conductor-vegetation contact during strong wind events. The problem is formulated in the context of proactive power shutoff with a focus on transmission systems. The ignition mechanism involves the flashover (or sparkover) phenomenon caused by a displaced conductor coming close to nearby trees. With the data on vegetation configuration, conductor-vegetation interaction is examined through specific distance quantities. The failure mechanism is modeled as the first-excursion problem, and the limit state is proposed as encroachment into the pre-defined baseline clearance (i.e., MVCD) in the alongwind lateral direction. By means of an efficient analysis in the frequency domain, dynamic effects of TL displacement responses are derived from wind turbulence and structural characteristics. The probability of encroachment is estimated based upon random vibration theory, and the effects of varied clearances and wind intensities are also explored.

It is found that the mean wind load accounts for most of the conductor blown-out displacement, to which the contribution of insulator string sway is non-negligible. The dynamic response around the mean wind state is dominated by the background response, as resonant response is suppressed by considerable aerodynamic damping. As shown by their standard deviation (high c.o.v., and comparable to MVCD), the dynamic effects of the displacement response are non-negligible. The sensitivity analyses reveal that for the range of probability where these calculations are meaningful (i.e., neither $P_{\textrm{en},r}(0)=1$ nor $P_{\textrm{en},r}({T^{}_{0})}=0$), the encroachment probability is shown to be sensitive to vegetation clearance and wind intensity. The proposed approach can be used at any identified checking points by virtue of the finite element implementation, as illustrated by the two-span TL example. To illustrate the transition from local checking points to the study of a de-energizable unit (such as a branch), the modified RTS-GMLC benchmark system example is used. The encroachment probability of any span along a branch can be appreciably high even if the encroachment probability of each individual span therein is very small. These sensitivity analyses cover the most important factors affecting the problem, but several other studies could be performed to determine the influence of secondary factors. It is important to point out that the ability of performing these sensitivity studies leverages the fact that a mechanistic approach has been developed. The data-driven approaches available in the literature could not do these sensitivity analyses because the available data for each combination of factors is insufficient. However, as it is usually the case for probabilistic approaches applied to rare event, a global validation of the results against real-world events or experimental work is not possible. Instead, a step-by-step validation of the components of the proposed approach is presented, including the characterization of the wind stochastic process, the mathematical description of real-time vegetation clearance, the definition of limit state, and the calculation of the probability of first-excursion failure.

Wildfire is becoming a global threat under the background of climate change. Yet it is a relatively recent area of interest for civil engineering compared to other hazards (e.g., earthquakes, hurricanes). The main contribution of this paper is the proposed methodology for predicting powerline ignition through systematic analysis of the conductor dynamic response under high winds. As opposed to the purely data-driven methods that base predictions on historical ignition records, the proposed approach is efficient, informative, and flexible in accommodating various combinations of wind loading, structure and vegetation. In particular, the calculated encroachment probability incorporates the influence of the event duration which is an important factor in weighing shutoff decisions. However, there are several points that need further attention. First, the overall approach and the accuracy of its results highly depend on the availability and accuracy of the input data, including those related to the electric facilities, vegetation, weather, etc. In California these data are being collected systematically and thoroughly (Bob Bell, Manager, Transmission Vegetation Management Dept., Pacific Gas & Electricity, personal communication, 2020), but this may not apply to all regions at risk of wildfires. Second, the two examples included in the manuscript have illustrative purposes, with some simplified characteristics (single conductor, symmetric structural geometry, assumed constant vegetation clearance). For a complex real-world transmission network, repeated calculations are needed for each different conductor-vegetation-weather setting. Third, the encroachment into MVCD (or conductor-vegetation contact) is just the initiating event in the chain that may or may not lead to powerline-induced wildfires. Given the current knowledge on flashover and the various factors affecting ignition, the probability of ignition given encroachment will require additional studies. Nevertheless, informed of the probability of encroachment, utility decision makers are able to leverage the encroachment-ignition knowledge gap as safety margin and make justifiable de-energization decisions.

## Supplementary Information

Supplementary Information.

## Data Availability

All data, models, or code that support the findings of this study are available from the corresponding author upon reasonable request.
