# Supplementary material for: Predicting wildfire ignition induced by dynamic conductor swaying under strong winds
Source: Sci Rep. 2023 Mar 10;13:3998. doi: 10.1038/s41598-023-30802-w (PMC10006223; doi:10.1038/s41598-023-30802-w)
Supplement: Supplementary file 1 — Supplementary Information. [file 41598_2023_30802_MOESM1_ESM.pdf]

Predicting wildfire ignition induced by dynamic conductor swaying under strong winds – Supplementary information

Xinyue Wang[1] **and Paolo Bocchini**[1,*]

[1]Dept. of Civil and Environmental Engineering, ATLSS Engineering Research Center, Lehigh Univ., Bethlehem, 18015, USA.
[*]Email: paolo.bocchini@lehigh.edu

Static wind hazard map of California

Historical wind records from the Remote Automated Weather Stations (RAWS) network were used for this purpose. With a 3-sec sampling frequency, the RAWS network reports hourly wind measurements at 6.1 m above the ground level, including peak 3-sec wind gust, 10-min average wind speed, and other information[1]. All wind data were downloaded through the Mesonet API provided by the MesoWest program[2,3]. Supplementary Figure S1 summarizes the approach to create the wind hazard map.

In particular, the hourly peak 3-sec wind gust (out of all samples in one hour) was chosen for wind hazard description. After data cleaning, 295 stations with satisfying data were left. In view of the at-station irregularity and across-station variation of data distributions, a nonparametric kernel distribution was fit for each station in MATLAB[4]. The annual exceedance probability with respect to a given wind intensity can be computed from the hourly exceedance probability using the equation below:

$$P_{\mathrm{oe}}^{\mathrm{a}} = 1 - (1 - P_{\mathrm{oe}}^{\mathrm{h}})^{8760} \,. \tag{S1}$$

where $P_{\mathrm{oe}}^{\mathrm{h}}$ and $P_{\mathrm{oe}}^{\mathrm{a}}$ are hourly exceedance probability and annual exceedance probability, respectively. The return period (T_{r}) of the event exceeding a certain intensity is related to $P_{\mathrm{oe}}^{\mathrm{a}}$ by Eq. (S2). Thus, the extreme wind gust with a 20-year return period can be computed inversely with $P_{\mathrm{oe}}^{\mathrm{a}} = 5\%$. Finally, at-site extremes (295 points) were interpolated to create a continuous map utilizing the ArcGIS Geostatistical Analysis extension[5].

$$T_{\mathrm{r}} = \frac{1}{P_{\mathrm{oe}}^{\mathrm{a}}} \,. \tag{S2}$$

By assuming spatial homogeneity of the wind data, the Kriging method was chosen for spatial interpolation because of its robustness and simplicity. However, this approach is affected by the poor density and uneven distribution of RAWS stations, which contrasts with the sensitivity of wind to local conditions. Therefore, it is important to note that there is lower confidence in the interpolated surface than at-station values.

References

1. NWCG (National Wildfire Coordinating Group). *Interagency Wildland Fire Weather Station Standards & Guidelines* (Boise, ID: NWCG, 2014).

2. Synoptic Data PBC. Mesonet API. Accessed January 07, 2023. https://developers.synopticdata.com/mesonet/.

3. University of Utah. MesoWest Data. Accessed January 07, 2023. https://mesowest.utah.edu/.

4. MATLAB. *9.8.0.1359463 (R2020a)* (The MathWorks Inc., Natick, Massachusetts, 2020).

5. Johnston, K., Ver Hoef, J. M., Krivoruchko, K. & Lucas, N. *Using ArcGIS geostatistical analyst*, vol. 380 (Esri Redlands, 2001).

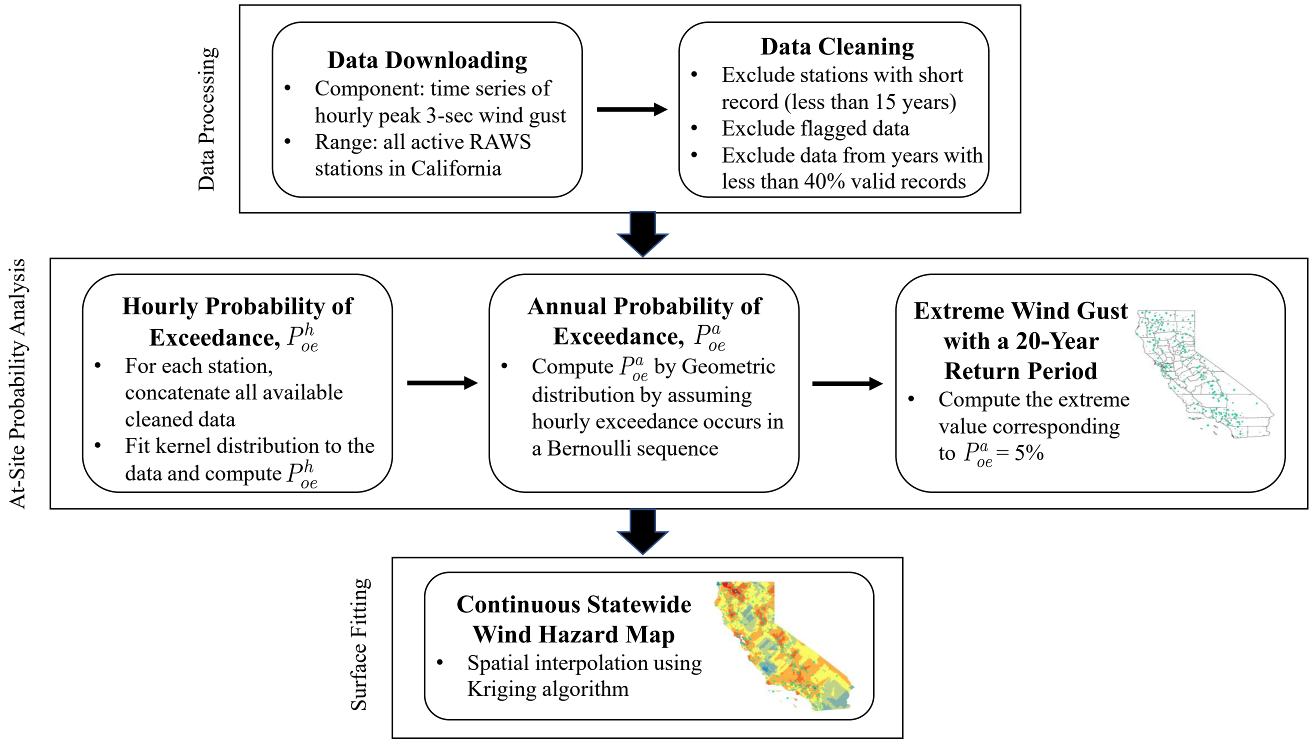

Fig. S1. Hazard map creation process.
